# Individual Optimal Attentional Strategy in Motor Learning Tasks Characterized by Steady-State Somatosensory and Visual Evoked Potentials

**DOI:** 10.3389/fnhum.2021.784292

**Published:** 2022-01-04

**Authors:** Takeshi Sakurada, Masataka Yoshida, Kiyoshi Nagai

**Affiliations:** ^1^Department of Robotics, College of Science and Engineering, Ritsumeikan University, Shiga, Japan; ^2^Major in Advanced Mechanical Engineering and Robotics, Graduate School of Science and Engineering, Ritsumeikan University, Shiga, Japan

**Keywords:** focus of attention, motor learning, individual differences, steady-state somatosensory evoked potentials, steady-state visual evoked potentials, sensory cortex

## Abstract

Focus of attention is one of the most influential factors facilitating motor performance. Previous evidence supports that the external focus (EF) strategy, which directs attention to movement outcomes, is associated with better motor performance than the internal focus (IF) strategy, which directs attention to body movements. However, recent studies have reported that the EF strategy is not effective for some individuals. Furthermore, neuroimaging studies have demonstrated that the frontal and parietal areas characterize individual optimal attentional strategies for motor tasks. However, whether the sensory cortices are also functionally related to individual optimal attentional strategy remains unclear. Therefore, the present study examined whether an individual’s sensory processing ability would reflect the optimal attentional strategy. To address this point, we explored the relationship between responses in the early sensory cortex and individuals’ optimal attentional strategy by recording steady-state somatosensory evoked potentials (SSSEP) and steady-state visual evoked potentials (SSVEP). Twenty-six healthy young participants first performed a motor learning task with reaching movements under IF and EF conditions. Of the total sample, 12 individuals showed higher after-effects under the IF condition than the EF condition (IF-dominant group), whereas the remaining individuals showed the opposite trend (EF-dominant group). Subsequently, we measured SSSEP from bilateral primary somatosensory cortices while presenting vibrotactile stimuli and measured SSVEP from bilateral primary visual cortices while presenting checkerboard visual stimuli. The degree of increasing SSSEP response when the individuals in the IF-dominant group directed attention to vibrotactile stimuli was significantly more potent than those in the EF-dominant individuals. By contrast, the individuals in the EF-dominant group showed a significantly larger SSVEP increase while they directed attention to visual stimuli compared with the IF-dominant individuals. Furthermore, a significant correlation was observed such that individuals with more robust IF dominance showed more pronounced SSSEP attention modulation. These results suggest that the early sensory areas have crucial brain dynamics to characterize an individual’s optimal attentional strategy during motor tasks. The response characteristics may reflect the individual sensory processing ability, such as control of priority to the sensory inputs. Considering individual cognitive traits based on the suitable attentional strategy could enhance adaptability in motor tasks.

## Introduction

The focus of attention is one of the most influential motor performance factors (Wulf et al., 2010). Previous studies investigated the effects of two distinct attentional strategies for motor learning: internal focus (IF) and external focus (EF). In the IF strategy, performers direct their attention to body movements, whereas performers direct their attention to movement outcomes in the EF strategy. Most previous studies on motor learning have found that the EF strategy improves motor performance compared to the IF strategy in many types of motor tasks such as basketball free-throw shooting (Zachry et al., 2005), volleyball serves (Wulf et al., 2002), golf pitch shots (Wulf and Su, 2007), and dart-throwing (Lohse et al., 2010; Wulf, 2013). The advantage of the EF strategy has been explained by the constrained-action hypothesis (Wulf et al., 2001); when individuals try to control their body movements actively, the attentional strategy corresponding to the IF strategy disrupts automatic processes for motor control. However, the disruption can be avoided when the attention is directed farther away from the body. This hypothesis is also supported by empirical findings such as facilitating high-frequency movement adjustments (McNevin et al., 2003) and efficient electromyography (EMG) activities during motor tasks (Zachry et al., 2005). For instance, when basketball players were asked to perform free throws under either the EF condition (directing their attention to the rim of the basket) or the IF condition (directing their attention to wrist flexion), the EF strategy was associated with not only an improvement in shooting accuracy but also a reduction in EMG activity in the biceps and triceps muscles. These findings suggest that the EF strategy enhanced fine and automatic motor control by reducing “noise” in the motor system associated with muscle activities (Zachry et al., 2005).

Although many previous studies have shown the advantage of the EF strategy, individual factors can weaken the advantage (Perkins-Ceccato et al., 2003; Emanuel et al., 2008). For instance, high-skilled golfers (mean handicap: 4) performed better under the EF than the IF condition. However, low-skill golfers (mean handicap: 26) showed better performance under the IF strategy than the EF strategy during the pitch shot task (Perkins-Ceccato et al., 2003). Furthermore, our recent studies on healthy and stroke populations demonstrated that the optimal attentional strategy for motor tasks depends on individual sensory modality dominance for cognitive function such as motor imagery (Sakurada et al., 2016a,^2017^, 2019a). Specifically, the participants with visual imagery dominance showed better motor performance under the EF condition (EF-dominant). In contrast, participants with kinesthetic imagery dominance showed higher motor performance under the IF condition (IF-dominant). These findings suggest that the EF strategy did not always lead to better motor performance and it is necessary to identify the individual optimal attentional strategy to obtain the maximum motor performance.

The neural basis has also been examined along with the accumulation of behavioral evidence regarding the focus of attention (mainly supporting EF strategy’s advantages). Some neuroimaging studies reported distinct neural activity patterns under different attentional conditions. For example, during a hitting-key task, the EF strategy in which participants directed their attention to keys rather than the IF strategy directing attention to finger movement induced greater activity in the primary somatosensory and motor cortices (Zentgraf et al., 2009). Another study reported that neural activation in the left lateral premotor cortex, left primary somatosensory cortex, and intraparietal lobule was induced by switching attentional strategy between IF and EF during a finger movement task (Zimmermann et al., 2012). These studies suggest that the cognitive attentional strategy can affect neural activities in motor- and sensory-related areas.

On the other hand, the differences in the brain dynamics between IF-dominant individuals and EF-dominant individuals have not been fully understood. Regarding this point, we recently demonstrated that the neural dynamics in the frontal and parietal areas, which are important regions for attentional control (Corbetta and Shulman, 2002; Dosenbach et al., 2008; Hu et al., 2013; Jerde and Curtis, 2013; Kehrer et al., 2015) are involved in determining an individual’s optimal attentional strategy. Specifically, during a visuomotor learning task, the right dorsolateral prefrontal and right somatosensory association cortices showed lower activity under the individual optimal attentional strategy. These findings suggest that the optimal attentional strategy can facilitate efficient neural processing in the frontoparietal network to accelerate its motor learning effect (Sakurada et al., 2019b). Furthermore, individuals including healthy young, healthy elderly, and acute stroke individuals with an IF dominance showed higher left prefrontal activity than those with EF-dominant under the IF condition during a simple cyclic hand movement task (Sakurada et al., 2019a). These results suggest that the prefrontal dynamics reflect an individual’s ability to process internal body information, characterizing optimal attentional strategy. Taken together, although the frontal and parietal areas are one of the crucial regions for individual optimal attentional strategy, the involvements of other areas remain unclear.

Here, although the IF and EF strategies are traditionally defined as the difference in the attentional target (i.e., body movement or movement outcome), an alternative interpretation of this definition based on the difference in the targeted sensory modality may help clarify the difference in brain characteristics between the IF- and EF-dominant individuals. Specifically, since the IF is a strategy that focuses attention on body movements, internal body information such as tactile or somatosensory information is processed preferentially. On the other hand, since the movement outcomes targeted during the EF mainly exist in the external environment, it can be assumed that the sensory modality to be processed preferentially is visual information. Indeed, the framework represents the brain’s various cognitive and motor functions based on the paired sensory modalities between internal and external body information. Examples of these kinds of modalities would be kinesthetic and visual motor imagery (Solodkin et al., 2004; Guillot et al., 2009; Hétu et al., 2013), tactile and visual working memory (Savini et al., 2012; Luck and Vogel, 2013), intrinsic muscle and extrinsic visual coordinates for motor representation (Swinnen, 2002; Sakurada et al., 2016b), and internal self-paced closed skill and externally paced open-skill sports (Di Russo et al., 2010). Interestingly, we recently demonstrated the correlation between the individual optimal attentional strategy (IF or EF strategy) and their modality dominance of motor imagery (kinesthetic or visual motor imagery) (Sakurada et al., 2016a,^2017^, 2019a). Therefore, we can extend the framework to the focus of attention, and the difference between IF and EF strategies can be alternatively interpreted as the difference in the sensory modality processed during each strategy. Regarding the individual differences in the optimal attentional strategy, we can interpret that IF- and EF-dominant individuals are good at processing tactile/somatosensory and visual sensory information, respectively.

If the targeted sensory modality defines the difference between IF and EF strategies, it is expected that the responses in the sensory area will be different between the IF- and EF-dominant individuals. Since the responses of the sensory areas changes due to the top-down projection from the frontoparietal networks involved in attention control (Valenza et al., 2004; Corbetta et al., 2005; Vuilleumier et al., 2008), it is highly possible that individual differences in the optimal attentional strategy also influence the sensory area’s responses. In this study, in order to examine the relationship between individual optimal attentional strategy and the characteristics of the sensory areas, we focused on the oscillatory electroencephalogram (EEG) responses in the sensory areas; steady-state somatosensory evoked potentials (SSSEP) and the steady-state visual evoked potentials (SSVEP). SSSEP can be observed in the primary somatosensory cortex when individuals receive vibrotactile stimuli at a constant frequency. By contrast, SSVEP can be observed in the primary visual cortex during receiving flickering visual stimuli. Furthermore, both SSSEP and SSVEP amplitudes are enhanced by the attention and reflect top-down facilitation of early sensory processing (Giabbiconi et al., 2004, 2007; Kim et al., 2007; Kashiwase et al., 2012), so the SSSEP/SSVEP is one of the brain signal suitable for quantifying the individual attention ability to sensory inputs. Based on these characteristics of SSSEP and SSVEP, we hypothesized that individuals with IF-dominant show strong attentional modulation of SSSEP, and conversely, those with EF-dominant have a strong SSVEP attentional modulation. To test our hypothesis, this study examined whether neural activities in the somatosensory and visual areas reflect an individual optimal attentional strategy for motor learning tasks. We first classified participants into IF- and EF-dominant individuals by comparing motor learning effects under the IF and EF conditions. Subsequently, based on this classification, we assessed differences in neural activity in the sensory areas between individuals with IF- and EF-dominant by recording SSSEP and SSVEP.

## Materials and Methods

### Participants

Twenty-six healthy participants (mean age ± SD, 22.4 ± 1.0 years; 3 females, 23 males) were recruited from the students at Ritsumeikan University. All participants were right-handed as assessed by the Edinburgh Handedness Inventory (laterality 91.9 ± 12.6) (Oldfield, 1971). This study was conducted in accordance with the Declaration of Helsinki and approved by the Institutional Review Board at Ritsumeikan University. All participants provided written informed consent before participation. In addition, each participant completed the following experimental protocol in a single day, including a motor learning task aiming to evaluate the individual optimal attentional strategy and EEG recording task to compare SSSEP and SSVEP responses.

### Motor Learning Task for Evaluating the Individual Optimal Attentional Strategy

#### Experimental Setup

**FIGURE 1:**
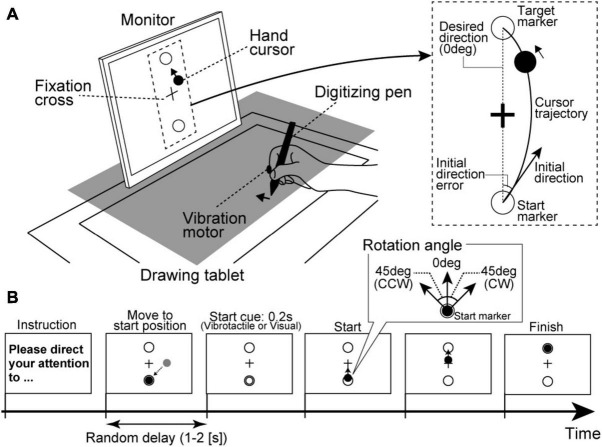
Experimental setup in the motor learning task. **(A)** A rack to hide a participant’s right hand is illustrated as a gray square. The participants control the hand cursor while looking at the fixation cross on the front monitor. **(B)** Trial procedure. Regarding the start cue, most trials presented sensory stimuli relevant to the attention target as the consistent trials (i.e., vibrotactile stimuli under the IF condition or visual stimuli under the EF condition) in order to properly guide the participants’ attention.

Each participant was seated on a chair and asked to hold a digitizing pen on a drawing tablet (Intuos4 PTK-1240/K0, Wacom, Japan) with their right hand. As shown in Figure 1A, an LCD monitor (size: 31.0 cm × 41.0 cm) for visual stimulus presentation was vertically placed 30 cm from a participant’s face. Because a cloth and small rack hid the participants’ right hand, they could not directly see their right hand during the experimental tasks. Furthermore, their head position was fixed by a chin rest. All visual stimuli on the monitor were programmed in MATLAB (MathWorks, Inc., Natick, MA, United States) using the Cogent Toolbox (University College London, London, United Kingdom). The position of the digitizing pen tip was recorded using the Cogent Toolbox with sampling at 60 Hz. The monitor displayed a hand cursor (filled black circle in Figure 1) as real-time visual feedback reflecting the participant’s hand movement. For instance, when a participant moved their hand to diagonally forward left direction on the tablet, the hand cursor synchronously moved to the upper left direction on the monitor. The participants were instructed to move their hands with their shoulder and elbow joints and not to move their fingers to control the hand cursor. The monitor also displayed a fixation cross at the center and two markers as start and target positions for reaching movements (open circles in Figure 1). The distance between the start and target markers on the monitor was 12 cm. The hand cursor moved 1.0 cm on the monitor for a 1.0-cm hand movement, so the drawing tablet’s desired amplitude of the reaching movement was 12 cm. A vibration motor presenting vibrotactile stimuli was attached to the index fingertip of the right hand.

#### Procedure

Regarding experimental conditions, we introduced IF and EF conditions. Under the IF condition, participants were instructed to direct their attention to their hand movements itself covertly. By contrast, participants were instructed to direct their attention to the hand cursor movements on the monitor under the EF condition. Furthermore, as the standard instruction among both conditions, they were asked to fixate the fixation cross on the monitor during the reaching movements.

Regarding the procedure of each trial, before starting a trial, the monitor displayed experimental instructions to remind participants of the attentional strategy (IF or EF) to be used during the reaching movements. The participants firstly needed to move the hand cursor to the start marker position. Then, after a random delay period (1–2 s), the sensory stimulus was briefly presented (0.2 s) as a movement start cue. Specifically, vibrotactile stimulus from vibration motor (tactile start cue) or a color flickering of the hand cursor (visual start cue) was presented. The participants were instructed to start a reaching movement as quickly as possible in reaction to a start cue. They were also instructed to control the hand cursor straight from the start marker to the target marker. Each trial ended 1 s after the hand cursor reached the target marker (Figure 1B).

Here, we defined the trial type according to the movement start cue in each attention condition. For example, under the IF condition requiring attention to hand movements, trials with the tactile start cue were defined as consistent trials (i.e., both attention target and start cue stimulus target were related to participants’ hands). In contrast, those with the visual start cue were defined as inconsistent trials (i.e., attention target was participants’ hand movement, and start cue stimulus target was hand cursor). By contrast, under the EF condition, trials with a visual start cue and with tactile start cue were defined as consistent trials (i.e., both attention target and start cue stimulus target were hand cursor) and inconsistent trials (i.e., attention target was hand cursor and start cue stimulus target was related to participants’ hand), respectively. The consistent trials had the role of guiding the participants’ attention to the instructed target (hand movement or hand cursor) under each condition.

The reaching task consisted of two sessions, and the IF and EF conditions were randomly assigned for each participant’s 1st and 2nd sessions. Each session had three phases: baseline, learning, and wash-out. In the baseline phase, the participants firstly performed ten consistent trials without visuomotor rotation (i.e., the hand cursor moved contingently according to the participant’s hand movement; visuomotor rotation angle = 0°). Then, the participants performed 40 trials (35 consistent trials and five inconsistent trials, the inconsistent trials were randomly inserted among the consistent trials) as the learning phase. In the learning phase, the hand cursor movement was rotated by 45° from the origin (start marker position) in either clockwise (CW) or counterclockwise (CCW) direction relative to the participant’s actual hand movement. The CW and CCW settings were randomly assigned to the IF and EF conditions. Half of the participants performed the reaching movements under the IF condition with the CW rotation and the EF condition with the CCW rotation. The other participants performed the reaching movements under the IF condition with the CCW rotation and the EF condition with the CW rotation. In addition, participants were required to correctly modify their hand movements to control the hand cursor in a straight trajectory. Finally, the participants performed ten consistent trials without visuomotor rotation as the wash-out phase. The baseline, learning, and wash-out phase trials were successively run without interruption, and participants were not informed in advance about the CW/CCW disturbance.

#### Analysis

##### Reaction Time

To confirm whether the participants correctly directed their attention to the hand movement itself (IF condition) or to the hand cursor (EF condition), we analyzed the RT to the tactile and visual start cues in the learning phase. Although the tactile start cue did not directly reflect the hand movement itself, which is the attention target of the IF condition, the vibrotactile stimulus was effective in leading the participant’s attention to the hand motor state. In other words, if participants correctly apply the IF strategy, it is expected that the responses to the tactile start cue presented to the body part performing the reaching movement will be shortened. While, since the visual start cue matched the attention target of the EF condition (i.e., hand cursor as a movement outcome) if participants correctly apply the EF strategy, the response to the visual start cue will be directly shortened. Our previous studies have also confirmed the effectiveness of linking such simple sensory inputs (vibrotactile and visual stimuli) with the IF and EF strategies (Sakurada et al., 2016a, 2019a,b). Therefore, in each trial, we defined the RT as the delay from the start cue presentation to the instant where tangential hand velocity exceeded 50 mm/s. We excluded RTs faster than 150 ms or slower than 1,500 ms (Ratcliff, 1993; Hultsch et al., 2002). The participants were considered to correctly direct their attention when the mean RT satisfied one of the following two equations.


(1) $$\left(RT_{IF}^{T}<RT_{EF}^{T}\right)\cap \left(RT_{EF}^{V}<RT_{IF}^{V}\right)$$

(2) $$\left(RT_{IF}^{T}<RT_{IF}^{V}\right)\cap \left(RT_{EF}^{V}<RT_{EF}^{T}\right)$$

Here, the superscripts *T* and *V* denote the modality of the start cue (*T*, Tactile; *V*, Visual), and the subscripts *IF* and *EF* denotes the attention condition. Thus, eqs. 1, 2 represent the situation where RT is faster in consistent trials than inconsistent trials.

##### Motor Performance and Attention Dominance

In each trial, the movement initiation was detected when the tangential hand velocity exceeded 50 mm/s, and the movement end was defined as when tangential hand velocity fell to 50 mm/s after the tangential peak velocity. Then, we calculated the initial direction error as the motor performance index. The initial direction was the direction of the initial hand velocity vector, connecting the start marker position and the hand cursor position on 100 ms after the movement initiation. Thus, initial direction error was defined as the angular difference between the directions of the visual target marker and the initial direction (Figure 1A, right panel). Note that in each participant, we defined the initial direction error in the visuomotor rotational direction in the learning phase as the positive direction. For instance, in a participant, when CW rotation was set for the IF condition, deviation to the right is a positive initial direction error. Similarly, when CCW rotation was set for the EF condition, deviation to the left is a positive value. To evaluate the degree of visuomotor learning under the IF and EF conditions, we focused on the after-effect, movement errors generated by unexpectedly removing the displacement of the visual hand cursor position after the learning phase (i.e., sudden removal of the visuomotor rotation in this task), indicating the learning a new transformation from the visual input to motor output in the brain (Krakauer, 2009). The after-effect in each condition was calculated based on the mean absolute initial direction error among the first to third trials of the wash-out phase. Additionally, the individual attentional strategy was determined by a difference value of the after-effect sizes between the IF and EF conditions. Specifically, we subtracted the after-effect under the IF condition from that under the EF condition. Because the size of the after-effect reflects the degree of learning effect for visuomotor rotation in the current motor task (i.e., large after-effect indicates strong motor learning effect), we defined the IF-dominant group as participants who showed larger after-effect under the IF condition than the EF condition (i.e., the difference of the after-effect is a negative value), and the EF-dominant group as participants who showed a larger after-effect under the EF condition than the IF condition (difference of the after-effect is a positive value), respectively.

#### Statistical Analysis

RTs were assessed by two-way repeated-measures analysis of variance (ANOVA) with “condition” (IF or EF) and “trial type” (consistent or inconsistent) as within-subject factors. In addition, to evaluate the degree of motor learning, a two-way ANOVA was applied to the after-effect size with “group” (IF- or EF-dominant) as a between-subjects factor and “condition” (IF or EF) as a within-subject factor. We used a significance threshold of *p* < 0.05 (two-tailed) for all tests.

### Steady-State Somatosensory Evoked Potentials and Steady-State Visual Evoked Potentials Recording

#### Experimental Setup

**FIGURE 2:**
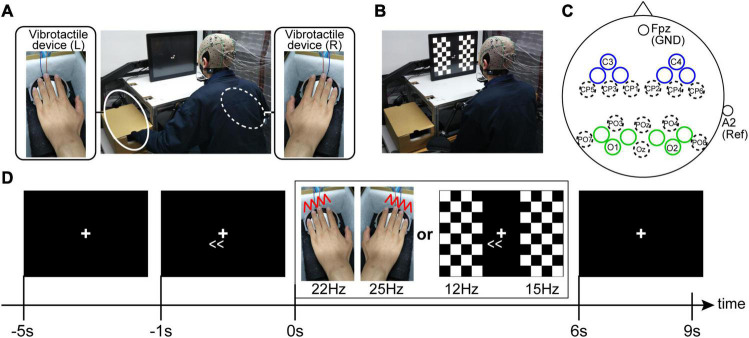
Experimental setup for the SSSEP recordings **(A)** and the SSVEP recordings **(B)**. During the SSVEP recording, the participants were instructed to put their hands on ones’ knees. **(C)** EEG electrode setup. Electrodes were placed in each bilateral sensory areas (blue and green circles indicate the electrode positions for SSSEP and SSVEP recording, respectively). The unlabeled blue and green circles are the recording electrode positions located at the midpoint of the electrode positions defined by the 10-10 extension coordinate system. **(D)** Trial procedure. The participants were instructed to sustain their attention in the indicated direction (left or right) during the stimulus presentation.

Each participant was seated on a chair in a dimly lit room, and a chin rest fixed the participants’ head position. The distance from the monitor to participants’ eyes was 30 cm. When recording SSSEPs, we prepared a self-build vibrotactile device. First, sine waves were output from Arduino, and then the amplified sine waves generated vibration of diaphragms on the device (vibration amplitude was about 2 mm). Because the most effective frequency range of vibrotactile stimulus for SSSEP is approximately 20–30 Hz (Tobimatsu et al., 1999; Ahn et al., 2016), we applied 22-Hz and 25-Hz vibrotactile stimuli for the left and right fingertips (index, middle, ring, and little fingers), respectively. In order to have constant contact pressure among the fingertips, bilateral fingertips were fixed on the rigid edge of the device (Figure 2A). When recording SSVEPs, the same LCD monitor used in the motor learning task (size: 31.0 cm × 41.0 cm, refresh rate: 60 Hz) showed pattern reversal stimuli with checkerboards. The checkerboard stimuli on the monitor were controlled by MATLAB using the Cogent Toolbox with 60 Hz. The left and right checkerboards were composed of 8 vertical × 4 horizontal squares (each size: 3.8 cm × 3.8 cm), respectively, and the white and black colors in each checkerboard stimuli were reversed at a specified frequency. The visual angles from the fixation cross to the center of the bilateral checkerboard stimuli were ±20°. Because the most effective frequency range of visual stimulus for SSVEP is approximately 10–20 Hz (Pastor et al., 2003), we applied 12-Hz and 15-Hz pattern reversal stimuli for the left and right visual fields, respectively (Figure 2B).

#### Electroencephalogram Data Acquisition

Electroencephalogram signals were detected by placing twenty Ag/AgCl active electrodes mounted in an elastic cap, according to the 10-5 EEG coordinate system. Specifically, for recording SSSEP, we used the EEG signals from the electrodes around the left and right primary somatosensory cortexes (blue circles in Figure 2C). By contrast, for recording SSVEP, we used the EEG signals from the electrodes around the left and right primary visual cortexes (green circles in Figure 2C). The ground was located at Fpz, and the reference was the right earlobe. The EEG signals were recorded with an OpenBCI Cyton+Daisy Biosensing Boards (OpenBCI, United States) with a sampling frequency of 1 kHz. Circuit impedance was kept below 20 kΩ for all electrodes. Furthermore, to check horizontal eye movements during the EEG recordings, two disposable electrodes were attached to the bilateral tails of the eyes, and the OpenBCI system also recorded electrooculography (EOG) signals.

#### Procedure

The EEG recording task consisted of four sessions. SSSEP and SSVEP recordings were assigned to the first two sessions in random order, and then these recordings were assigned again in reversed order in the last two sessions to reduce the order effect in each participant.

Each session had ten trials. A fixation cross was continually displayed in the center of the monitor during the trials and the participants were instructed to gaze at it. Furthermore, the participants were instructed to push a footswitch by their foot when they were ready to start a trial in each trial. Four seconds after pushing the footswitch, an arrow indicating the direction of attention (leftward or rightward) was displayed on the monitor, and the participants were asked to direct their attention to the indicated direction covertly. Then, the sensory stimuli (vibrotactile stimuli in the SSSEP recording sessions or checkerboard stimuli in the SSVEP recording sessions) were presented for 6 s from 1 s after the appearance of the arrow. Finally, after an interval of 3 s, a trial returns to the waiting phase to start the subsequent trial (Figure 2D). Regarding the attention direction, each session had five trials presented with a right-directional arrow (i.e., right-attention condition) and five with a left-directional arrow (i.e., left-attention condition). The order of the left- and the right-attention conditions were randomized in each session.

#### Analysis

Data processing was performed offline using custom-written MATLAB scripts. In the SSVEP recordings, the visual angles from the fixation cross to the edges of the left and right checkerboard stimuli (i.e., right edge of the left checkerboard and left edge of the right checkerboard) were five degrees. Thus, eye movement with five degrees or more means that a participant directly saw the visual stimulus at the central visual field. To eliminate this scenario, the trial was excluded from the analysis when eye movement was detected from EOG signals, specifically when an EOG voltage change corresponding to eye movement of 5° or more. To eliminate the influence of eye movements, the exclusion criterion was also applied to the SSSEP recordings. After excluding the trials with eye movement, the EEG signals for 5 s (i.e., from 1 to 6 s after stimulus onset) were filtered with a two-order 5–55 Hz bandpass filter in each trial. Then, to evaluate the SSSEP and SSVEP responses under the left- and right-attention conditions, we calculated frequency spectrum density by a fast Fourier transform applying to the filtered EEG signals. Here, when the participants directed their attention to either the left or right sensory stimuli, an increase in the SSSEP/SSVEP response can be observed on the contralateral hemisphere (Giabbiconi et al., 2007; Kim et al., 2007; Bardouille et al., 2010; Kashiwase et al., 2012). Conversely, a decreasing or no change of SSSEP/SSVEP response can be observed on the ipsilateral hemisphere. Based on the attention modulation characteristics of SSSEP/SSVEP, we defined the “modulation index (*MI*)” representing the degree of SSSEP/SSVEP response change depending on the participant’s covert attention by the eqs. (3–6):


(3) $$MI_{S1}^{L}=\left(PP_{25Hz}^{AttR}-PP_{25Hz}^{AttL}\right)-\left(PP_{22Hz}^{AttR}-PP_{22Hz}^{AttL}\right)$$

(4) $$MI_{S1}^{R}=\left(PP_{22Hz}^{AttL}-PP_{22Hz}^{AttR}\right)-\left(PP_{25Hz}^{AttL}-PP_{25Hz}^{AttR}\right)$$

(5) $$MI_{V1}^{L}=\left(PP_{15Hz}^{AttR}-PP_{15Hz}^{AttL}\right)-\left(PP_{12Hz}^{AttR}-PP_{12Hz}^{AttL}\right)$$

(6) $$MI_{V1}^{R}=\left(PP_{12Hz}^{AttL}-PP_{12Hz}^{AttR}\right)-\left(PP_{15Hz}^{AttL}-PP_{15Hz}^{AttR}\right)$$

Here, the superscripts L and R on the left-hand side denote the hemisphere (left hemisphere: L, right hemisphere: R), and the subscripts S1 and V1 on the left-hand side denote the targeted sensory area (primary somatosensory cortex: S1, primary visual cortex: V1). Thus, for instance, eq. (3) represents the MI in the left primary somatosensory cortex. PP is spectrum peak power at stimulus frequency in the left- or right-attention conditions. The superscripts AttL and AttR on the right-hand side denote the trial condition (AttL, left-attention condition; AttR, right-attention condition), and the subscripts on the right-hand side denote the targeted frequency to calculate the spectrum peak power. Therefore, MI is an index that summed the increase in the SSSEP/SSVEP response elicited by attention-directed stimuli and the amount of decrease in the SSSEP/SSVEP response elicited by non-attention stimuli in each sensory area. Note that, eqs. (3, 4), and eqs. (5, 6) are equivalent. After calculating the MI for each EEG electrode, the mean MI values among the three electrodes for each sensory area were used as the representative value.

#### Statistical Analysis

In order to clarify the relationship between the individual optimal attentional strategy observed in the first motor learning task and the attention modulation in SSSEP/SSVEP response, we compared the MI between IF- and EF-dominant groups by Wilcoxon rank-sum tests. In addition, Pearson’s correlation coefficients were calculated for the inter-subject variability of the degree of attention dominance and MI. We used a significance threshold of *p* < 0.05 (two-tailed) for all tests.

## Results

### Motor Learning Task

#### Reaction Time

We excluded trials in which RT did not meet our criterion (<150 or >1,500 ms). In total, 0.5% of consistent trials and 0.1% of inconsistent trials were excluded under the IF condition, and 0.3% of consistent trials and 1.2% of inconsistent trials were excluded under the EF condition.

Comparison of RTs revealed a significant main effect of trial type [*F*(1,25) = 108.92, *p* = 1.34 × 10^–10^, η_p_^2^ = 0.81], but the main effect of the condition [*F*(1,25) = 0.25, *p* = 0.62, η_p_^2^ = 0.01] and the two-way interaction [*F*(1,25) = 0.65, *p* = 0.43, η_p_^2^ = 0.025] did not reach statistical significance. These statistical results revealed that RTs for consistent trials were significantly faster than those for inconsistent trials [IF condition: 385.5 ± 53.7 SD ms (consistent trials) vs. 503.7 ± 90.4 SD ms (inconsistent trials). EF condition: 375.4 ± 60.4 SD ms (consistent trial) vs. 505.8 ± 108.6 SD ms (inconsistent trials)] and suggests that all participants correctly directed their attention according to experimental instructions.

#### Optimal Attentional Strategy

**FIGURE 3:**
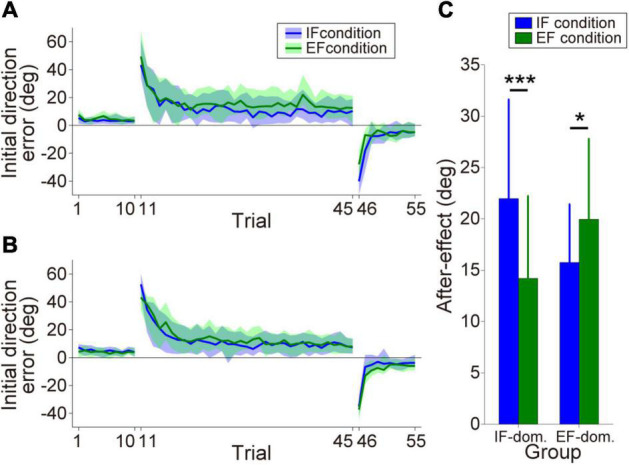
Motor performance in the motor learning task. **(A,B)** Initial direction error transitions in the IF-dominant group **(A)** and EF-dominant group **(B)**, respectively. Blue and green lines indicate the mean initial direction errors under IF and EF conditions, respectively (note: only the consistent trials are plotted). The lighter colored regions around the mean lines denote the standard deviation. **(C)** After-effect sizes in each dominant group. Blue and green bars indicate after-effects under IF and EF conditions, respectively. Error bars denote the standard deviation. *p < 0.05, ***p < 0.001.

By comparing the size of the after-effect between the IF and EF conditions, we classified the participants into the IF-dominant group (*n* = 12) or EF-dominant group (*n* = 14). Figures 3A,B show the initial direction error changes as the consistent trials progressed (IF-dominant group, Figure 3A; EF-dominant group, Figure 3B). The hand cursor did not deviate from the desired straight trajectory in the baseline phase without visuomotor rotation (1st–10th trials). However, when the hand cursor movement was rotated during the learning phase (11th–45th trials), the initial direction error markedly increased and gradually decreased. At the end of the learning phase, initial direction error reached a plateau indicating that the participants in both groups successfully adapted to the visuomotor rotation. Finally, when the visuomotor rotation was removed in the wash-out phase (46th–55th trials), the participants showed large initial direction errors in the opposite direction (i.e., after-effect). The after-effects in each dominant group are shown in Figure 3C. Analysis of after-effect size revealed a significant two-way interaction of group × condition [Group: *F*(1,24) = 0.023, *p* = 0.88, η_p_^2^ = 0.001, Condition: *F*(1,24) = 2.25, *p* = 0.15, η_p_^2^ = 0.086, Interaction: *F*(1,24) = 25.34, *p* = 0.000038, η_p_^2^ = 0.51]. Post-hoc analysis with Bonferroni correction for the group × condition interaction revealed that the IF-dominant group showed significantly larger after-effect under the IF condition [*p* = 0.00017, simple main effect test] and the EF-dominant group showed significantly larger after-effect under the EF condition [*p* = 0.016, simple main effect test].

### Steady-State Somatosensory Evoked Potentials and Steady-State Visual Evoked Potentials Responses

Trials excluded based on EOG data comprised 3.2% of all trials.

**FIGURE 4:**
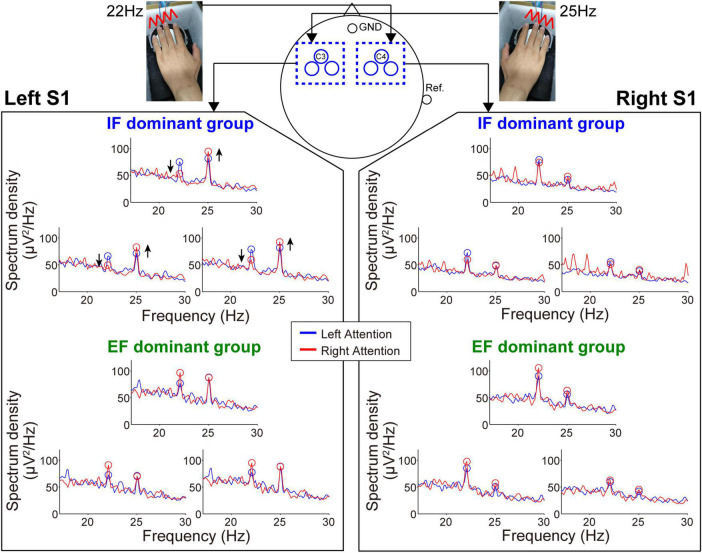
Frequency spectrum in the SSSEP recording session. The vibrotactile stimuli presented to the fingertips mainly induced the SSSEP responses in the primary somatosensory area on the contralateral hemisphere. Blue and red lines indicate the spectrum under the left- and right-attention conditions, respectively. Bule and red circles indicate the peak points at stimulus frequencies. Attention modulation was strongly observed in the IF-dominant group as shown by the arrows in the spectrum of the left hemisphere.

**FIGURE 5:**
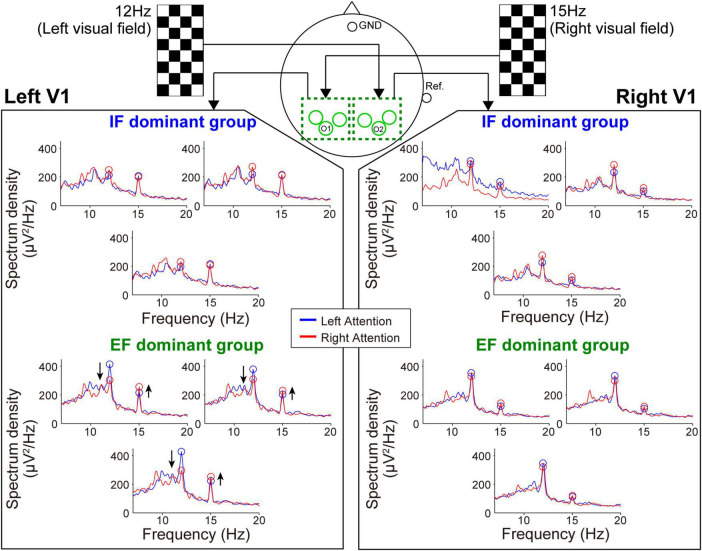
Frequency spectrum in the SSVEP recording session. Attention modulation was strongly observed in the EF-dominant group as shown by the arrows in the spectrum of the left hemisphere.

Figures 4, 5 show the mean spectrum power of SSSEP from the primary somatosensory cortex and SSVEP from the primary visual cortex, respectively. The spectrum peaks at the stimulation frequencies indicate the induced SSSEPs and SSVEPs. First, we focus on the change in the peak power by comparing the left- and right-attention conditions in SSSEP recording (Figure 4). As a typical trend, the response from the left hemisphere in the IF-dominant group, the intensity at 25 Hz, was more substantial under the right-attention condition than under the left-attention condition. Conversely, the response intensity at 22 Hz was weaker when they directed attention to the right stimuli. On the other hand, no clear attention modulation was detected in the EF-dominant group, even in the left hemisphere. Regarding the right hemisphere, both IF- and EF-dominant groups did not show marked attention modulation.

Next, during the SSVEP recording, the tendency of attention modulation was reversed between both groups (Figure 5). Specifically, the responses from the left hemisphere tended to strengthen the 15 Hz spectrum peak and weaken the 12 Hz spectrum peak under the right-attention condition only in the EF-dominant group. Furthermore, as in the case of SSSEP recording, no marked attention modulation was observed in the right hemisphere in both groups.

**FIGURE 6:**
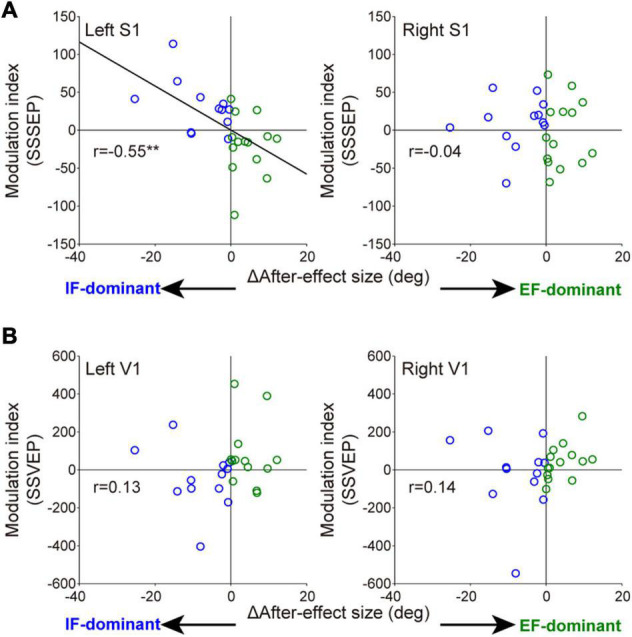
Relationships between individual optimal attentional strategy and the attention modulation observed in SSSEP **(A)** and SSVEP **(B)**. The horizontal axis indicates the individual attention dominance calculated by the difference of after-effect size. Positive and negative values indicate IF- and EF-dominant, respectively. Modulation index (MI) on the longitudinal axis is averaged value among three electrodes in each sensory area. Blue and green circles indicate the individuals with IF dominance and those with EF dominance, respectively. Significant correlation was found in the left somatosensory cortex (right S1: *r* = −0.04, *p* = 0.83. left V1: *r* = 0.13, *p* = 0.54. right V1: *r* = 0.14, *p* = 0.51). **p < 0.01.

Wilcoxon rank-sum tests for the mean MI among three electrodes in each sensory area revealed that the degree of SSSEP attention modulation in the left primary somatosensory cortex was significantly stronger in the IF-dominant group than in the EF-dominant group (IF-dominant group, 31.1 ± 34.3 SD; EF-dominant group, −19.1 ± 39.0 SD; *p* = 0.001). By contrast, the EF-dominant group showed a significantly stronger SSVEP attention modulation in the left visual cortex than the IF-dominant group (IF-dominant group, −46.0 ± 157.4 SD; EF-dominant group, 136.7 ± 285.7 SD; *p* = 0.048). No significant group difference was observed in the right hemisphere (SSSEP: IF-dominant group, 10.1 ± 33.7 SD; EF-dominant group, −4.3 ± 44.1 SD; *p* = 0.52. SSVEP: IF-dominant group, −21.7 ± 201.3 SD; EF-dominant group, 43.0 ± 95.7 SD; *p* = 0.40). Figure 6 shows the individual attention dominance quantified in the first motor learning task and MI in the second EEG recording task. Again, there was a significant correlation between the two indices in SSSEP responses; individuals with more robust IF dominance had larger SSSEP attention modulation in the left hemisphere (r = −0.55, *p* = 0.003).

## Discussion

Consistent with our previous studies (Sakurada et al., 2016a,^2017^, 2019a,b), the EF strategy did not always improve motor performance in about half of the participants. These results support our previous evidence that individual optimal strategy can facilitate motor learning effect. Furthermore, in this study, we demonstrated the involvement of sensory areas in the individual optimal attentional strategy. As expected, individuals with IF-dominant and those with EF-dominant had distinct neural dynamics to the sensory inputs. Therefore, these findings imply that we can alternatively interpret the difference between IF and EF strategies based on the attended sensory modality (i.e., tactile vs. visual) instead of the traditional definition based on the difference of the attention target (i.e., body movement vs. movement outcome). Here, we discuss the relationship between attention control and sensory processing.

### Individual Differences in Optimal Attentional Strategy Based on Sensory Processing

Individual sensory modality dependency in the early sensory areas characterizes optimal attentional strategy during motor tasks. Specifically, the current findings indicated that higher abilities to directing attention to tactile and visual sensory inputs lead to the IF- and EF dominance, respectively. Regarding the attention modulations in SSSEP and SSVEP, we observed both increases at the attended stimulus-response and a decrease at the unattended stimulus-response in the current results. When the IF-dominant individuals received multi-frequency vibrotactile stimuli or the EF-dominant individuals received multi-frequency visual stimuli, suggesting that the individual superior cognitive ability to both enhancement and suppression is the basis of the optimal attentional strategy during motor tasks. The relationship between the attention process and responses in the sensory areas observed in this study can fit the findings of a previous study that the early sensory areas are enhanced by the attention process (Poghosyan and Ioannides, 2008). Furthermore, from the viewpoint of attention, directing attention to a specific sensory stimulus modality controls the priority for the sensory inputs and contributes to facilitating perceptual sensitivity to the attended sensory information (Rahnev et al., 2011; Itthipuripat et al., 2014). Such attention processes have a crucial role in efficiently processing vast amounts of sensory inputs.

On the other hand, it is also important to suppress unnecessary (i.e., irrelevant) stimuli in tasks to perform sensory processing efficiently. It is well known that the sensory gating system contributes to filtering out irrelevant stimuli from the external environment in the brain. For instance, when paired-pulse stimuli with short inter-stimulus intervals are presented, a sensory gating phenomenon is observed; a specific oscillation response to the second stimulus is strongly reduced relative to that elicited by the first stimulus (Wiesman et al., 2017; Wiesman and Wilson, 2020). Such suppression process to sensory inputs also contributes to optimizing the neural resources available for behaviorally relevant neural computations (Cromwell et al., 2008). Furthermore, this filtering for suppressing sensory inputs is influenced by attention (Ishii et al., 2019; Wiesman and Wilson, 2020), and oscillation in the alpha band is considered to be important for the attentional suppression mechanism (Foxe and Snyder, 2011). Thus, attention to a proper sensory modality for individuals may enhance the motor learning effect by the combination of selecting the task-relevant sensory signals and suppressing the task-irrelevant sensory signals (Pestilli et al., 2011).

Although it is desired that SSSEP and SSVEP can characterize individual optimal attentional strategy, the task-dependency on the effectiveness of the current approach is unclear. In other words, we currently applied a lab-based visuomotor task based on simple upper limb movement with a low degree of freedom, but many previous studies investigating the focus of attention used various kinds of practical motor tasks with a high degree of freedom, such as basketball free-throw shooting (Zachry et al., 2005), golf pitch shots (Wulf and Su, 2007), and dart-throwing (Lohse et al., 2010; Wulf, 2013). It is essential to clarify whether the SSSEP- and SSVEP-based approach is also useful for other practical motor tasks as a test bed to characterize the individual cognitive trait. Following this point, in our preliminary results, we confirmed that the individual optimal attentional strategy in a simple visuomotor task is also an effective attentional strategy for improving another practical motor task with a high degree of freedom (data not shown). Therefore, the current SSSEP- and SSVEP-based evaluations may be widely effective without task-dependency.

### Large Variation in Ability to Direct Attention to Internal Body Information

Individual cognitive ability to process tactile or somatosensory information rather than visual information is a more important brain characteristic that forms the optimal attentional strategy during motor tasks. This finding is consistent with our previous reports (Sakurada et al., 2017, 2019a). In our daily life, since we actively direct attention to visual targets frequently, it is assumed that everyone can direct attention to the external space. On the other hand, the experience of actively and voluntarily directing attention to one’s internal body sensory information will widely vary among individuals. For instance, individual sports experience has a significant influence. Specifically, since the internal body information has a crucial role in sports such as gymnastics or swimming, which strongly requires closed skills, individuals with such closed skill sports experience tend to have an IF-dominant (Sakurada et al., 2016a). Thus, it is interpreted that sports experience is one of the influential factors that increased individual differences in the attention ability to internal body information and formed individual dynamics in the somatosensory area. Furthermore, regarding the type of attention function evaluated in this study, the cognitive process reflected by SSVEP can be regarded as spatial attention to the external space. On the other hand, we can assume that the individual attention evaluated by SSSEP is the body-specific attention directed to near own body part(s) (Reed et al., 2006, 2010; Aizu et al., 2018). That is, the current findings might imply that the ability of body-specific attention rather than spatial attention to the external space has a large variance among individuals.

### Sensory Stimulus Frequency for Quantifying Individual Attention Ability

The current study demonstrated that SSSEP and SSVEP are useful brain signals to evaluate individual attention ability based on sensory modality dependency. In this study, we applied the effective frequency band with the strongest response in SSSEP and SSVEP (Tobimatsu et al., 1999; Pastor et al., 2003; Ahn et al., 2016). However, given the relationship between brain function and neural oscillations, a more useful frequency band may quantify individual attention ability. Indeed, attention is associated with various ranges of neural oscillations, such as alpha and gamma bands. For instance, when directing one’s attention to the left or right visual field, alpha band power in the ipsilateral posterior parietal cortex increases (Thut et al., 2006; Rihs et al., 2007). Thus, the increased alpha oscillations relate to an inhibitory attentional mechanism to task-irrelevant information in the unattended visual field.

On the other hand, directing attention to the somatosensory stimulus resulted in an increased gamma-band in the primary somatosensory cortex (Dockstader et al., 2010), attention to the right median nerve caused the gamma-band synchronization (Gobbelé et al., 2002). Taken together, because many previous studies reported that alpha band oscillations correlate negatively with attention and performance (i.e., inhibition/suppression of sensory processing) and gamma-band oscillation correlate positively with attention and performance (i.e., facilitation of sensory processing), it may be reasonable to use higher-frequency stimuli when assessing an individual’s ability to direct attention to specific sensory inputs. To verify the frequency dependence can promote the development of individual difference evaluation methods.

### Laterality of Attention Modulation

Although the current results suggest that the left hemisphere has neural dynamics that reflect individual optimal attentional strategy, we need to interpret this laterality carefully. First, hand dominance can influence attention modulation in the left hemisphere. Since all the participants in this study were right-handed, it is considered that they were more sensitive to the tactile or somatosensory stimuli on the right hand or to the visual stimuli in the right visual field where the right hand is present. The attentional bias to the right body or the right external space may have caused strong SSSEP and SSVEP attention modulation in the left hemisphere. Furthermore, the frequency of visual stimuli can induce laterality. Especially regarding SSVEP attention modulation, the peak of SSVEP amplitude in occipital regions was observed at 15 Hz (Pastor et al., 2003). These strong response characteristics may elicit large attention modulation in the left hemisphere processing 15 Hz-visual stimuli in this study. Note that, attention function itself exhibits lateralization (superior in the right hemisphere) (Corbetta et al., 2000; Müri et al., 2002; Shulman et al., 2010), it is also possible that such functional laterality may be an influential factor. Further investigations are required to clarify this point. For instance, we may prove this point by collecting data on left-handed participants or exploring the frequency characteristics of SSSEP and SSVEP other than the frequency adopted in this study. If the individual’s dominant hand affects the laterality of attention modulation, the laterality suggests a functional aspect of attention control (i.e., intrinsic factor), whereas if there is a stimulation frequency dependency, it would simply suggest that the frequency characteristics of SSSEP and SSVEP responses lead to the laterality (i.e., extrinsic factor).

In addition to the neurophysiological factors, the influence of the experimental setup cannot be excluded. For example, previous studies reported that SSSEP and SSVEP and their attention modulation could also be observed in the frontal area where activities from the sensory area are projected (Herrmann, 2001; Giabbiconi et al., 2007; Adler et al., 2009; Pang and Mueller, 2014). Therefore, when the SSSEP or SSVEP is measured with the reference on the frontal area, the attention modulation component observed in the sensory area might be attenuated. Therefore, the reference electrode was placed on the right earlobe where the cortical signal was not measured to avoid this concern in the current study. However, under the current electrode montage, since the reference electrode was located near the right hemisphere, it might be challenging to detect the attention modulation of SSSEP and SSVEP in the right hemisphere. Therefore, as a better experimental setup, we need to adopt a different montage with the reference electrodes placed on the bilateral ear lobes in the future.

## Conclusion

We demonstrated that individual differences in optimal attentional strategy during motor tasks reflect individual characteristics of sensory processing in the early sensory areas. Specifically, individuals with IF-dominant had a high ability to direct their attention to body movements and implicitly direct attention to internal body information, such as tactile or somatosensory inputs related to body movements. By contrast, individuals with EF-dominant had a high ability to direct their attention to external body information such as visual inputs. These findings indicated that the dynamics of wide neural circuits, including the frontoparietal network responsible for attention control and the sensory areas, have an essential role in characterizing individual optimal attentional strategy. Thus, identifying individual differences in cognitive function depending on sensory modality will contribute to developing tailor-made protocols that maximize motor learning effects and adaptability.

## Data Availability Statement

The raw data supporting the conclusions of this article will be made available by the authors, without undue reservation.

## Ethics Statement

The studies involving human participants were reviewed and approved by the Institutional Review Board at Ritsumeikan University. The participants provided their written informed consent to participate in this study.

## Author Contributions

TS conceived and designed the experiment, developed the experimental system, wrote the draft of the manuscript, and supervised the study. MY performed the participant experiments. TS and MY analyzed the data. All authors contributed to the discussion of the results and read and approved the final manuscript.

## Conflict of Interest

The authors declare that the research was conducted in the absence of any commercial or financial relationships that could be construed as a potential conflict of interest.

## Publisher’s Note

All claims expressed in this article are solely those of the authors and do not necessarily represent those of their affiliated organizations, or those of the publisher, the editors and the reviewers. Any product that may be evaluated in this article, or claim that may be made by its manufacturer, is not guaranteed or endorsed by the publisher.
